# Ovary and embryo proteogenomic dataset revealing diversity of vitellogenins in the crustacean *Gammarus fossarum*

**DOI:** 10.1016/j.dib.2016.07.045

**Published:** 2016-07-28

**Authors:** Judith Trapp, Jean-Charles Gaillard, Arnaud Chaumot, Olivier Geffard, Olivier Pible, Jean Armengaud

**Affiliations:** a Irstea, Unité de Recherche MALY, Laboratoire d’écotoxicologie, CS70077, F-69626 Villeurbanne, France; b CEA-Marcoule, DSV/IBITEC-S/SPI/Li2D, Laboratory “Innovative technologies for Detection and Diagnostic”, BP 17171, F-30200 Bagnols-sur-Cèze, France

**Keywords:** Crustacean, Embryogenesis, Non-model organism, Oogenesis, Proteogenomics Reproduction, Vitellogenins

## Abstract

Ovaries and embryos from sexually mature *Gammarus fossarum* were sampled at different stages of the reproductive cycle. The soluble proteome was extracted for five biological replicates and samples were subjected to trypsin digestion. The resulting peptides were analyzed by high resolution tandem mass spectrometry with a LTQ-Orbitrap XL instrument. The MS/MS spectra were assigned with a previously described RNAseq-derived *G. fossarum* database. The proteins highlighted by proteogenomics were monitored and their abundance kinetics over the different stages revealed a large panel of vitellogenins. Criteria were i) accumulation during oogenesis, ii) decrease during embryogenesis, iii) classified as female-specific, and iv) sequence similarity and phylogenetic analysis. The data accompanying the manuscript describing the database searches and comparative analysis (“*High-throughput proteome dynamics for discovery of key proteins in sentinel species: unsuspected vitellogenins diversity in the crustacean Gammarus fossarum*” by Trapp et al. [1]) have been deposited to the ProteomeXchange via the PRIDE repository with identifiers PRIDE: PXD001002.

**Specifications Table**

**Table:** 

Subject area	*Environmental biology*
More specific subject area	*Amphipod comparative proteogenomics*
Type of data	*Figure*
How data was acquired	*Data-dependent acquisition of tandem mass spectra using a LTQ-Orbitrap-XL mass spectrometer (Thermo).*
Data format	*Raw*
Experimental factors	For each female, ovaries or embryos were dissected under stereomicroscope magnification, immediately frozen in liquid nitrogen and stored at −80 °C until needed. Proteins were extracted and analyzed by shotgun proteogenomics. Five biological replicates were performed for each stage. Five molt stages were analyzed: post-molt stage A and B, inter-molt stage C1, inter-molt stage C2, pre-molt stage D1, and pre-molt stage D2. Accordingly, five different embryo development stages were analyzed.
Experimental features	*The 50 soluble protein extracts were briefly run on SDS-PAGE, followed by in-gel trypsin proteolysis. Tryptic peptides were analyzed by nanoLC-MS/MS and spectra were assigned with a RNA-seq derived protein sequence database.*
Data source location	CEA-Marcoule, DSV-Li2D, Laboratory “Innovative technologies for Detection and Diagnostics”, BP 17,171, F-30200 Bagnols-sur-Cèze, France
Data accessibility	Data is within this article and deposited to the ProteomeXchange via the PRIDE repository with identifier PRIDE: PXD001002.

**Value of the data**

• The data represent the largest repertoire of proteins involved in reproduction established from a crustacean based on 50 samples from various stages of the reproductive cycle.
• Because gammarids are considered as sentinel organisms with great potential in the field of ecotoxicology and more specifically freshwater health monitoring, these data represent a useful resource for potential toxicological biomarkers [2].
• The data have been used to characterize the diversity of vitellogenins in amphipods. As described in detail in the companion manuscript [1], this diversity is rather unusual and calls for additional functional characterization of this crucial family of proteins.

## 1 Data

**Fig. 1:**
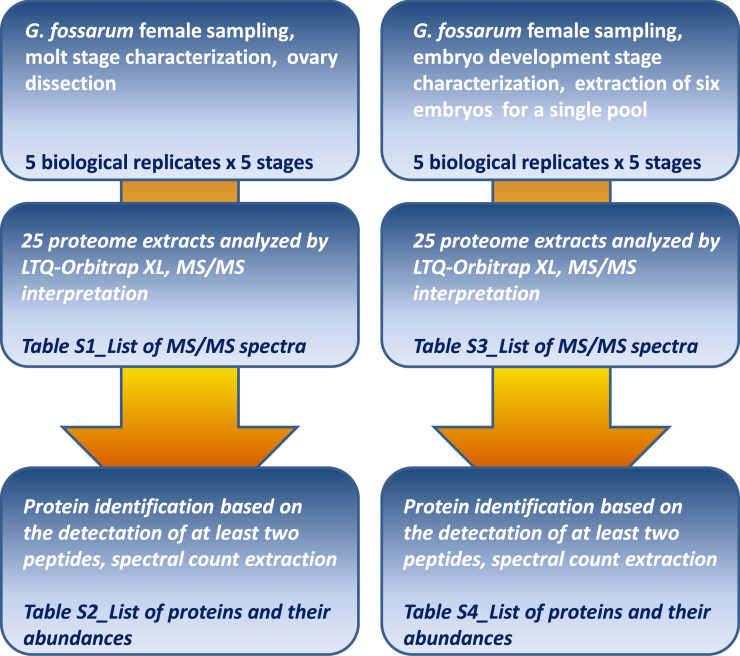
Flowchart of experiments, data processing and refined data tables.

Fig. 1 shows the schematic flowchart of experiments, data processing and interpreted results that were presented in four large tables and published recently [1]. The proteome data from five independent biological replicates per stages (5 different oocyte molt stages and 5 different embryo development stages), i.e. from 50 proteome samples, were assigned to trypsic peptides against the *G. fossarum* RNAseq derived database described by Trapp et al. [3] following a proteogenomic strategy [4], [5], [6]. The Deposited data comprised the 50 raw files, the protein sequence database, and the interpreted files.

## 2 Experimental design, materials and methods

### 2.1 Sampling of animals and preparation of biological samples

Female *G. fossarum* amphipods were collected as described [1]. Based on limb inter-tegmental change criteria, molt stages of female gammarids were classified into five different categories: post-molt stages A and B, inter-molt stage C1, inter-molt stage C2, pre-molt stage D1, and pre-molt stage D2, as previously described [7]. Accordingly, five different embryo development stages were also delineated. For each female, six embryos were collected from the ventral pouch and the ovaries were excised under stereomicroscope magnification as described by Lacaze et al. [8]. For each stage, five biological replicates were performed, immediately frozen in liquid nitrogen and stored at −80 °C until needed. Proteins were processed as previously described [1], [9].

### 2.2 Tandem mass spectrometry

Peptide identification was performed by nanoLC-MS/MS with a LTQ-Orbitrap XL hybrid mass spectrometer (ThermoFisher) coupled to an UltiMate 3000 LC system (Dionex-LC Packings) [10], [11]. Peptides were resolved on a nanoscale C18 PepMapTM 100-capillary column (LC Packings) prior to injection into the ion trap mass spectrometer as previously described [12]. Full-scan mass spectra were measured from *m*/*z* 300 to 1800 with the LTQ-Orbitrap XL mass spectrometer in data-dependent mode with a scan cycle initiated with a full scan of high mass accuracy in the Orbitrap followed by MS/MS scans in the linear ion trap on the three most abundant ions.

### 2.3 Data interpretation and monitoring of proteome dynamics

MS/MS spectra were assigned against the GFOSS protein database, created from RNA-seq data acquired on *G. fossarum*. This database comprises 1,311,444 entries totaling 289,084,257 amino acids. Molecular ion peak lists were extracted as described previously by Christie-Oleza et al. [13]. Peptide assignation with MASCOT and protein validation were done with the parameters described previously [1] The number of spectra recorded per protein (spectral counts) was extracted from the spectra-to-peptide data set for each protein and each experimental condition. The proteome dynamics along the different stages was assessed with the TrendQuest module of the PatternLab program [14].
